# Early post-discharge mortality in CAP: frequency, risk factors and a prediction tool

**DOI:** 10.1007/s10096-022-04416-5

**Published:** 2022-02-08

**Authors:** Verena Glöckner, Mathias W. Pletz, Gernot Rohde, Jan Rupp, Martin Witzenrath, Grit Barten-Neiner, Martin Kolditz, for the CAPNETZ Study Group, M. Dreher, C. Cornelissen, W. Knüppel, D. Stolz, N. Suttorp, P. Creutz, M. Witzenrath, A. Mikolajewska, A. le Claire, M. Benzke, T. Bauer, D. Krieger, M. Prediger, S. Schmager, M. Kolditz, B. Schulte-Hubbert, S. Langner, G. Rohde, O. Degen, A. Hüfner, C. Hoffmann, T. Welte, J. Freise, G. Barten-Neiner, M. Nawrocki, I. Fuge, J. Freise, J. Naim, W. Kröner, T. Illig, N. Klopp, C. Kroegel, A. Moeser, M. Pletz, B. Schleenvoigt, C. Bahrs, D. Drömann, P. Parschke, K. Franzen, J. Rupp, N. Käding, M. Wouters, K. Walraven, D. Braeken, C. Spinner, H. Buschmann, A. Zaruchas, T. Schaberg, I. Hering, W. Albrich, F. Waldeck, F. Rassouli, S. Baldesberger, M. Panning, M. Wallner

**Affiliations:** 1 Division of Pulmonology, Medical Department I, University Hospital Carl Gustav Carus of TU Dresden, Fetscherstr. 74, Dresden, 01307 Germany; 2 Institute of Infectious Diseases and Infection Control, Jena University Hospital/Friedrich-Schiller-University, Jena, Germany; 3 Medical Department I, Department of Respiratory Medicine, Goethe University Hospital, Frankfurt/Main, Germany; 4 Department of Infectious Diseases and Microbiology, University Hospital Schleswig-Holstein, Lübeck, Germany; 5 German Center for Infection Research (DZIF), Partner Site Hamburg-Lübeck-Borstel, Giessen, Germany; 6 Department of Infectious Diseases and Pulmonary Medicine, and Division of Pulmonary Inflammation, Charité – Universitätsmedizin Berlin, Berlin, Germany; 7 Biomedical Research in Endstage and Obstructive Lung Disease Hannover (BREATH), Member of the German Center for Lung Research (DZL), Munich, Germany; 8 CAPNETZ STIFTUNG, Hannover, Germany; 9 German Center for Lung Research (DZL), Giessen, Germany

**Keywords:** Community-acquired pneumonia, Prognosis, Post-discharge, Mortality

## Abstract

There are few data on mortality after discharge with community-acquired pneumonia (CAP). Therefore, we evaluated risk factors for 30-day post-discharge mortality after CAP. We included all patients of the prospective multi-national CAPNETZ study between 2002 and 2018 with (1) hospitalized CAP, (2) survival until discharge, and (3) complete follow-up data. The study endpoint was death within 30 days after discharge. We evaluated risk factors including demographics, comorbidities, admission CAP severity, and laboratory values and treatment-related factors in uni- and multivariable analyses. A total of 126 (1.6%) of 7882 included patients died until day 30 after discharge, corresponding to 26% of all 476 deaths. After multivariable analysis, we identified 10 independent risk factors: higher age, lower BMI, presence of diabetes mellitus, chronic renal or chronic neurological disease (other than cerebrovascular diseases), low body temperature or higher thrombocytes on admission, extended length of hospitalization, oxygen therapy during hospitalization, and post-obstructive pneumonia. By addition these factors, we calculated a risk score with an AUC of 0.831 (95%CI 0.822–0.839, *p* < 0.001) for prediction of post-discharge mortality. Early post-discharge deaths account for ¼ of all CAP-associated deaths and are associated with patient- and CAP-severity-related risk factors. Additional studies are necessary to replicate our findings in independent cohorts. Study registration: NCT 02139163.

**Supplementary Information:**

The online version contains supplementary material available at 10.1007/s10096-022-04416-5.

## Introduction

Complications and short- or long-term mortality after hospital discharge because of community-acquired pneumonia (CAP) contribute significantly to the burden of this disease [1]. Whereas long-term mortality after CAP is predominantly comorbidity-related [2, 3], early (within 30 days) post-discharge complications might have potential for CAP-related causes susceptible to specific interventions. Recent population-based German data demonstrated a high mortality increase of 4.7% between in-hospital mortality (17.2%) and 30-day mortality (21.9%) in a cohort of 16.274 hospitalized patients with CAP [4]. A study from the USA (*n* = 3.000.000 2005–2015) also showed an alarmingly high 30-day post-discharge mortality of 8.2% for patients with pneumonia [5]. Other recent studies identified 30-day post-discharge mortality rates between 3 and 6% [6–10].

However, from large multicenter cohorts, there are very few data regarding risk factors for early post-discharge complications or mortality of patients that were hospitalized with CAP [7]. Accordingly, recent international guidelines on management of CAP provide almost no evidence-based recommendations for post-hospital care [11, 12]. In order to identify, initiate, and tailor optimized patient follow-up and structured discharge interventions to improve post-discharge prognosis, data on specific risk factors are necessary.

Therefore, the present study aimed to evaluate risk factors for early (within 30 days) post-discharge mortality in patients surviving hospitalization because of CAP from the CAPNETZ cohort.

## Methods

### Study cohort

Since 2001, the multi-national prospective CAPNETZ study (study registration NCT 02139,163) prospectively collects data an biosamples on patients with CAP in more than 40 local clinical centers in central Europe (https://www.capnetz.de/html/capnetz/lccs). A detailed description of the CAPNETZ methodology is given elsewhere [13]. Criteria for inclusion in CAPNETZ are age ≥ 18 years, a pulmonary infiltrate diagnosed by chest imaging, and at least one of the following criteria: history of fever (temperature ≥ 38.3 °C), cough, production of purulent sputum, or focal chest signs on auscultation. Exclusion criteria are acquired or therapeutically induced immune deficiency, active TB, or nosocomial acquisition of infection. All patients are followed up according to a standardized protocol for 180 days and all clinical parameters are stored in an electronic database. To evaluate outcome parameters, all patients or their relatives are contacted either personally or by phone for structured interviews. Written informed consent is obtained from every patient before inclusion in the study, and the study was approved by the local ethical committees of each participating center.

**Fig. 1:**
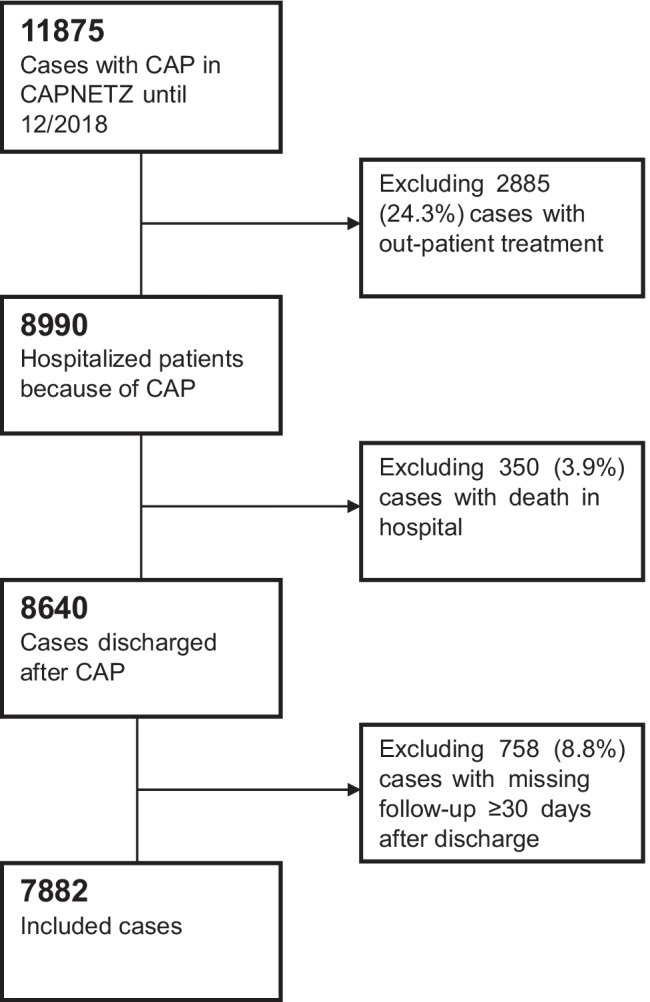
Flow chart on case selection

For the current study, we included all patients documented within the CAPNETZ cohort between 2002 and 2018 with (1) hospitalized CAP, (2) survival until discharge, and (3) complete follow-up data until at least 30 days after discharge. The resulting flowchart of the study cohort is depicted in Fig. 1.

### Definition of the endpoint and risk factors and statistical analysis

The primary endpoint was defined as death within 30 days after discharge from the hospital.

The evaluated risk factors regarding 30-day post-discharge mortality in the study population included a variety of demographic characteristics, pre-existing chronic comorbidities, laboratory values, vital parameters, microbiological results, and treatment-related factors documented within the CAPNETZ database. Missing values are clearly stated. CRB-65 and CURB-65 scores were calculated as described before [14]; missing values for respiratory rate were set as negative criterion for score calculation.

In the exploratory data analysis, the results were presented as median with the corresponding interquartile range (IQR). Continuous variables were analyzed using a non-parametric Mann–Whitney *U* test for two groups of continuous data to compare median values. For categorical variables, the Pearson chi-squared test was performed to analyze differences in proportions. To identify risk factors that are independently associated with 30-day post-discharge mortality, factors significantly associated (*p* < 0.05) after univariable analysis and with missing of less than 5% were entered into a multiple logistic regression model with stepwise forward selection (inclusion level 0.05, exclusion level 0.1). Results are presented as odds ratio (OR) with 95% confidence intervals (CI).

In an additional exploratory analysis, we evaluated a potential score for post-discharge mortality prediction by adding all risk factors independently associated after multivariable analyses. Categorical variables were handled as present or not for score calculation. Cut-offs of continuous variables significantly associated with 30-day post-discharge mortality were evaluated using receiver operating characteristic (ROC) analysis. The optimal cut-offs were determined by Youden-Index and its associated criterion. To evaluate diagnostic properties of the resulting score, we used ROC curves (AUC) and calculated sensitivity, specificity, positive/negative predictive value (PV), and positive/negative likelihood ratio (LR) with their 95% CIs. Additionally, we produced a Kaplan–Meier survival curve; comparison between the groups was performed by log-rank test.

For all evaluations, a *p*-value of ≤ 0.05 (two-sided) was considered statistically significant. Statistical analyses were performed with StatView 9.4 (SAS Institute Inc.) and SPSS Version 29 (IBM corp., Armonk, NY, USA).

## Results

### Patient characteristics and baseline risk factors

Of the 7882 patients included in our cohort study, the endpoint of death within 30 days after hospital discharge occurred in 126 (1.6%) patients. If this number is added to the 350 documented in-hospital deaths within the CAPNETZ cohort (Fig. 1), this corresponds to 26% of all 476 deaths in the study cohort until day 30 after discharge. For 103/126 (82%) deceased patients, data on the place of death were documented in the database: 58 (56%) died during another hospitalization within 30 days after discharge from the initial CAP-related hospitalization (4 on ICU), 24 (23%) died at home, 19 (18%) in a nursing home, and 2 (2%) during rehabilitation.

Table 1 depicts the demographic characteristics, pre-existing chronic comorbidities, vital parameters, laboratory results, and score values (CURB-65, CRB-65) on hospital admission in relation to the evaluated outcome 30-day post-discharge mortality. Demographic characteristics associated with the primary outcome parameter (with *p* < 0.05) in univariable analysis were higher age, lower BMI, smoking, residency in a nursing home, pre-existing long-term oxygenation therapy (LTOT), and pre-existing enteral nutrition. In univariable analysis, all except two (chronic liver disease and chronic respiratory disease) of the documented comorbidities were associated with 30-day post-discharge mortality. Regarding admission laboratory parameters, thrombocyte counts, hemoglobin, glucose, and urea were significantly associated with the endpoint. Additionally, pneumonia severity on admission as referenced by vital parameter abnormalities of respiratory rate, temperature, mental state, and the CRB-65 and CURB-65 scores were significantly associated with the outcome.

**Table 1:** Baseline characteristics of hospitalized CAP patients according to 30-day post-discharge survival status (bold, *p* < 0.05) (missing values are reported as available cases/all cases)

	Post-discharge mortality ≤ 30 days		*p*-value
	Deceased	Not deceased	
Total *n* (%)	126 (1.6%)	7756 (98.4%)	
*Demography*			
** Age (years), median (IQR)**	**77 (12)**	**66 (25)**	** < 0.001**
** Age ≥ 65 years, *n* (%)**	**111 (88.1)**	**4215 (54.3)**	** < 0.001**
** BMI, median (IQR) (7721/7882)**	**24.0 (5.13)**	**25.2 (6.7)**	** < 0.001**
Male, *n* (%)	84 (66.7)	4604 (59.4)	0.097
** Resident in a nursing home, *n* (%)**	**28 (22.2)**	**415 (5.4)**	** < 0.001**
** Smoker (last 12 months), *n* (%)**	**17 (13.5)**	**2286 (29.5)**	** < 0.001**
** Enteral nutrition, *n* (%)**	**7 (5.6)**	**91 (1.2)**	** < 0.001**
Chronic home ventilation, *n* (%)	0 (0.0)	30 (0.4)	0.484
** Long-term oxygen therapy (LTOT), *n* (%)**	**18 (14.3)**	**482 (6.2)**	** < 0.001**
Previous antibiotic therapy ≤ 4 weeks before hospital admission, *n* (%)	26 (20.6)	1834 (23.6)	0.430
Vaccination against influenza in previous 12 months, *n* (%) (7532/7882)	47 (43.5)	2712 (36.5)	0.135
*Comorbidities*			
** Chronic heart diseases other than chronic heart failure, *n* (%)**	**67 (53.2)**	**2813 (36.3)**	** < 0.001**
Chronic respiratory diseases, *n* (%)	44 (34.9)	2399 (30.9)	0.337
** Chronic heart failure, *n* (%)**	**51 (40.5)**	**1560 (20.1)**	** < 0.001**
** Diabetes mellitus, *n* (%)**	**39 (31.0)**	**1479 (19.1)**	**0.001**
** Chronic renal diseases, *n* (%)**	**32 (25.4)**	**781 (10.1)**	** < 0.001**
** Malignant tumor diseases, *n* (%)**	**25 (19.8)**	**768 (9.9)**	** < 0.001**
** Cerebrovascular diseases, *n* (%)**	**30 (23.8)**	**751 (9.7)**	** < 0.001**
** Other chronic neurological diseases, *n* (%)**	**24 (19.0)**	**491 (6.3)**	** < 0.001**
Chronic liver diseases, *n* (%)	2 (1.6)	222 (2.9)	0.393
*Vital parameters on hospital admission*			
** Confusion, *n* (%)**	**25 (19.8)**	**610 (7.9)**	** < 0.001**
** Body temperature ( °C), median (IQR) (7836/7882)**	**37.3 (1.4)**	**37.9 (1.7)**	** < 0.001**
** Respiratory rate (min**^**−1**^**), median (IQR) (7406/7882)**	**22 (8)**	**20 (6)**	**0.003**
** Respiratory rate ≥ 30 min**^**−1**^**, *n* (%)**	**19 (15.1)**	**712 (9.2)**	**0.024**
Heart rate (min^−1^), median (IQR) (7827/7882)	91 (24)	90 (21)	0.440
Systolic BP (mmHg), median (IQR) (7834/7882)	130 (36)	130 (29)	0.584
Diastolic BP (mmHg), median (IQR) (7829/7882)	70 (80)	75 (15)	0.371
BP < 90 mmHg (syst.) or ≤ 60 mmHg (diast.), *n* (%)	33 (26.2)	1618 (20.9)	0.145
*Laboratory results on hospital admission*			
Leucocytes (Gpt/l), median (IQR) (7779/7882)	13.33 (7.48)	12.10 (7.00)	0.072
** Hemoglobin (mmol/l), median (IQR) (7730/7882)**	**7.88 (1.51)**	**8.26 (1.43)**	** < 0.001**
** Thrombocytes (Gpt/l), median (IQR) (7646/7882)**	**275 (156)**	**237 (126)**	**0.003**
C-reactive protein (mg/l), median (IQR) (7719/7882)	88 (163.6)	108 (184.1)	0.318
Sodium (mmol/l), median (IQR) (7744/7882)	137 (6)	137 (5)	0.614
** Urea (mmol/l), median (IQR) (6945/7882)**	**7.79 (6.66)**	**5.70 (4.50)**	** < 0.001**
** Urea > 7 mmol/l, *n* (%) (6945/7882)**	**60 (54.1)**	**2426 (35.5)**	** < 0.001**
** Glucose (mmol/l), median (IQR) (7295/7882)**	**7.39 (3.02)**	**6.78 (2.62)**	**0.017**
*Scores*			
** CURB-65-Index, median (IQR) (6945/7882)**	**2 (2)**	**1 (2)**	** < 0.001**
0, *n* (%)	4 (3.6)	1918 (28.1)	
1, *n* (%)	29 (26.1)	2169 (31.7)	
2, *n* (%)	43 (38.7)	1821 (26.6)	
3, *n* (%)	27 (24.3)	776 (11.4)	
4, *n* (%)	5 (4.5)	141 (2.1)	
5, *n* (%)	3 (2.7)	9 (0.1)	
** CRB-65-Index, median (IQR)**	**1 (1)**	**1 (1)**	** < 0.001**
0, *n* (%)	6 (4.8)	2542 (32.8)	
1, *n* (%)	65 (51.6)	3533 (45.6)	
2, *n* (%)	45 (35.7)	1437 (18.5)	
3, *n* (%)	7 (5.6)	228 (2.9)	
4, *n* (%)	3 (2.4)	16 (0.2)	

### Microbiology

In 2103 patients (26.7%) of the study population, at least one pathogen was detected at hospitalization (Table 2). Two-third of infections in those 2103 patients with positive pathogen detection were caused by either *Streptococcus pneumoniae* (35%) and/or *Mycoplasma pneumoniae* (31.2%). Other pathogens detected were *Enterobacteriaceae* (13.2%), *Legionella* spp. (12.6%), *Haemophilus influenzae* (5.9%), *Staphylococcus aureus* (3.6%), *influenza* (5.2%), and other viruses (2.1%). Univariable analysis showed a higher rate of post-discharge mortality for infections with *Enterobacteriaceae* (*p* = 0.007) and *Staphylococcus aureus* (*p* = 0.001) (Table 2).

**Table 2:** Association of microbiological etiology with 30-day post-discharge mortality (more than one pathogen per patient possible) (bold *p* < 0.05)

	Post-discharge mortality ≤ 30 days		*p*-value
	Deceased	Not deceased	
No pathogen identified	88	5691	
Detection of pathogen, *n* (%)	38 (30.2)	2065 (26.6)	0.374
*Streptococcus pneumoniae*, *n* (%)	7 (18.4)	728 (35.3)	0.142
*Mycoplasma pneumoniae*, *n* (%)	11 (28.9)	645 (31.2)	0.867
***Enterobacteriaceae*, *n* (%)**	**10 (26.3)**	**267 (12.9)**	**0.007**
Legionella spp., *n* (%)	7 (18.4)	258 (12.5)	0.169
*Haemophilus influenzae*, *n* (%)	2 (5.3)	123 (6.0)	0.999
Influenza, *n* (%)	2 (5.3)	108 (5.2)	0.853
***Staphylococcus aureus*, *n* (%)**	**5 (13.2)**	**71 (3.4)**	**0.001**
Other viruses, *n* (%)	1 (2.6)	44 (2.1)	0.738

### Risk factors during hospital course of CAP

A number of parameters during the hospital course follow-up are documented within the CAPNETZ-database. Their associations with the outcome are presented in Table 3. We found a significant association with the endpoint for need of various kinds of respiratory support during CAP hospitalization as well as for length of hospital stay (LOS), occurrence of a pleural effusion, need of antibiotic treatment change, and for the final diagnosis of post-obstructive pneumonia.

**Table 3:** Characteristics during hospitalization of CAP-patients according to 30-day post-discharge survival status (bold *p* < 0.05)

	Post-discharge mortality ≤ 30 days		*p*-value
	Deceased	Not deceased	
**Length of hospitalization (LOS) in days, median (IQR)**	**14 (10)**	**10 (6)**	** < 0.001**
**Oxygen therapy anytime, *n* (%)**	**114 (90.5)**	**5370 (69.2)**	** < 0.001**
**New onset oxygen therapy during pneumonia, *n* (%)**	**96 (76.2)**	**4888 (63.0)**	**0.002**
**Oxygen therapy in follow up but not on admission to hospital, *n* (%)**	**16 (12.7)**	**514 (6.6)**	**0.007**
**New onset mechanical ventilation (non invasive or invasive) because of CAP, *n* (%)**	**18 (14.3)**	**548 (7.1)**	**0.002**
ICU admission, *n* (%)	7 (5.6)	354 (4.6)	0.597
Treatment with vasopressors, *n* (%)	2 (1.6)	55 (0.7)	0.248
**Post-obstructive pneumonia, *n* (%)**	**12 (9.5)**	**110 (1.4)**	** < 0.001**
**Pleural effusion anytime, *n* (%)**	**38 (30.2)**	**1520 (19.6)**	**0.003**
**Pleural effusion developing during hospitalization, *n* (%)**	**9 (7.1)**	**213 (2.7)**	**0.003**
**Change of antibiotic treatment during hospitalization, *n* (%)**	**60 (47.6)**	**3015 (38.9)**	**0.046**

### Multivariable regression analyses

After excluding variables with missing of more than 5% (glucose and urea), 7431/7882 of the cases (94.3%) including 110/126 patients meeting the endpoint were analyzed by multivariable regression analyses including all remaining parameters associated with post-discharge mortality after univariable analyses. Ten variables were independently associated with post-discharge mortality (Table 4).

**Table 4:** Multivariable regression analysis including all risk factors associated with post-discharge mortality after univariable analyses with less than 5% missings (exclusion of urea and glucose). A total of 7431/7882 cases (94.3%) with complete data for all included risk factors were analyzed. For directly associated variables (e.g., oxygen therapy anytime, new onset oxygen therapy during pneumonia, oxygen therapy in follow-up but not on admission), only the risk factor with the highest univariable OR was included in the model

	Post-discharge mortality ≤ 30 days		Cut-offs, for continuous variables determined by Youden Index
	OR (95%-CI)	*p*-value	
*Variables associated with p* < *0.05*			
Age	1.049 (1.032–1.067)	< 0.001	> 71 years
BMI	0.900 (0.863–0.938)	< 0.001	≤ 26
Diabetes mellitus	1.636 (1.066–2.510)	0.024	Present
Chronic renal diseases	2.080 (1.317–3.286)	0.002	Present
Other chronic neurological diseases	1.960 (1.135–3.384)	0.016	Present
Body temperature	0.768 (0.637–0.925)	0.005	≤ 38.2 °C
Thrombocyte counts	1.002 (1.001–1.004)	0.008	> 298 Gpt/l
Length of hospitalization	1.030 (1.017–1.042)	< 0.001	> 13 days
Oxygen therapy anytime	2.664 (1.371–5.177)	0.004	Present
Post-obstructive pneumonia	4.960 (2.344–10.496)	< 0.001	Present
*Variables not associated*			
Resident in a nursing home		0.083	
Smoker (last 12 months)		0.144	
Pre-existing enteral tube nutrition		0.132	
Pre-existing long term oxygen therapy		0.146	
Malignant disease		0.103	
Chronic heart failure		0.270	
Chronic heart diseases other than chronic heart failure		0.987	
Cerebrovascular diseases		0.059	
Confusion		0.145	
Respiratory rate		0.335	
Hemoglobin		0.596	
CRB-65 score		0.073	
* Staphylococcus aureus*		0.591	
* Enterobacteriaceae*		0.127	
New onset mechanical ventilation (non-invasive or invasive) because of CAP		0.186	
Pleural effusion developing during hospitalization		0.220	
Change of antibiotic treatment during hospitalization		0.338	

**Fig. 2:**
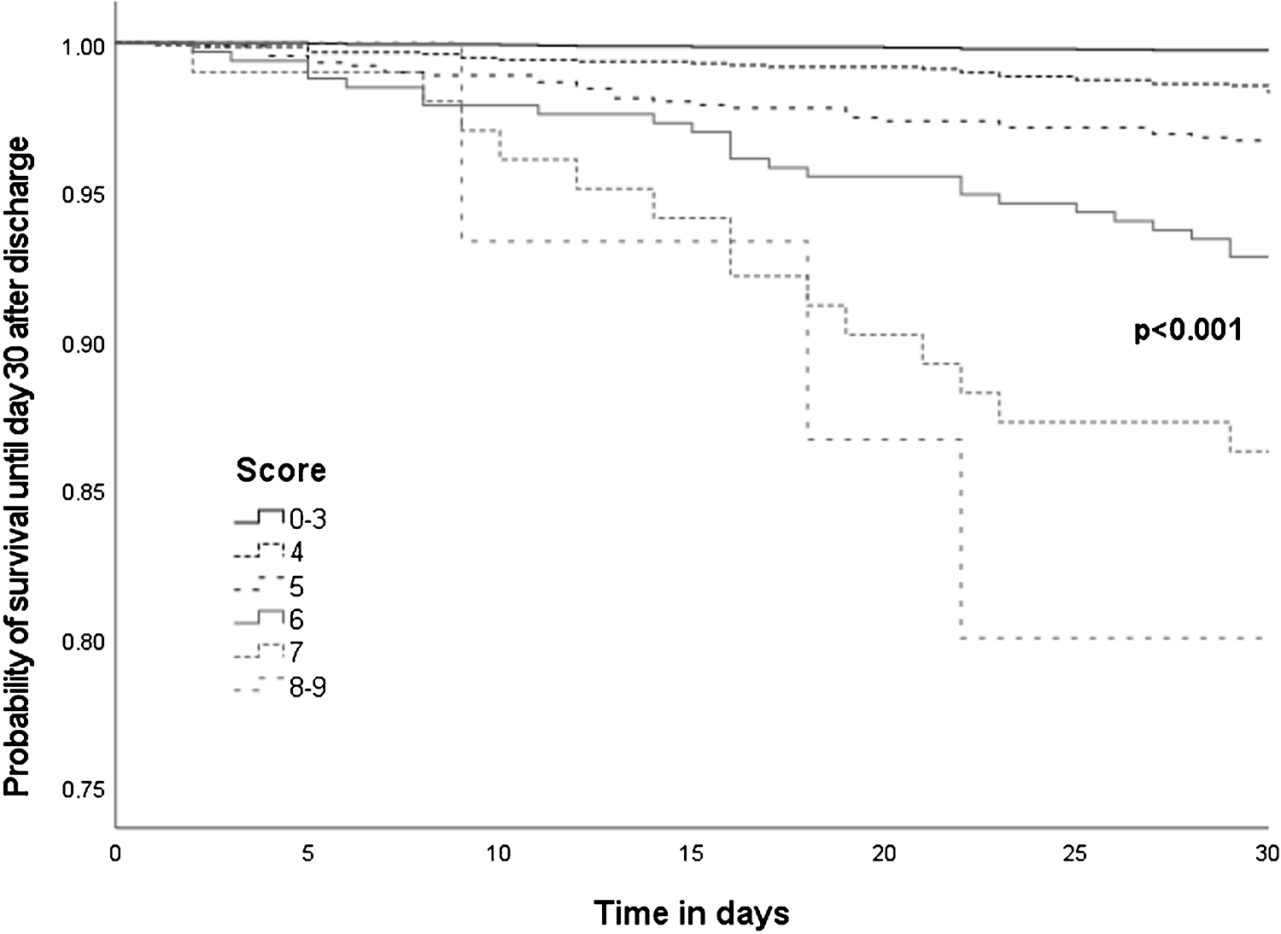
Kaplan–Meier analysis for prediction of post-discharge surrvival probability according to score values (scores of 0–3 and of 8–9 are grouped together)

By addition of each of the independently associated risk factors, we calculated a risk prediction score with values between 0 and 10 for 7449 patients with complete values for all 10 criteria (including 110/126 patients who met the primary endpoint). Frequency and proportion of 30-day post-discharge mortality according to defined score-values are shown in the supplementary e-Fig. 1. The score was significantly associated with post-discharge survival probability as demonstrated by Kaplan–Meier analysis (*p* < 0.001, Fig. 2). The score showed an area under the curve (AUC) of 0.831 (95% CI 0.822–0.839, *p*-value < 0.001) for predicting 30-day post-discharge mortality. The suggested optimal cut-off according to Youden Index was > 3 score points with a sensitivity of 90.0% ( 95% CI 82.8–94.9), a specificity of 60.9% (95% CI 59.8–62.0), a positive LR of 2.30 (2.1–2.5), and a negative LR of 0.16 (0.09–0.3). Detailed diagnostic properties for each cut-off of the score are depicted in the supplementary e-Table 1; the distribution of score values among the study population is shown in supplementary e-Fig. 2.

## Discussion

To understand the relationship between CAP and early post-discharge mortality and to identify a risk group to target intensified post-discharge-follow-up, we analyzed data from the large-scale well-characterized prospective multi-national CAPNETZ study. The main findings of our study are that (1) early post-discharge mortality accounts for 26% of all deaths occurring until day 30 after hospital discharge in hospitalized CAP, and (2) several risk factors were associated with early post-discharge mortality including demographic factors, comorbidities, admission laboratory and vital parameters and factors related to the hospital course of CAP.

In our study cohort, early post-discharge mortality of 1.6% was rather low compared to other recent cohorts which showed early post-discharge mortality rates between 3 and 8% [4, 6–10] and even up to 11% in a recent study from Turkey [15]. One plausible explanation might be that most studies included retrospective cohorts or population-based evaluations, whereas in CAPNETZ patients are prospectively recruited after a requested informed consent, which might introduce selection bias towards younger and healthier patients. This additionally is illustrated by the rather low hospital mortality rate within our cohort of 3.9% when compared to population based data of hospitalized CAP in Germany with hospital mortality rates of 12–17%, and is further reflected by the lower median age and lower rates of chronic comorbidities when compared to the population based cohorts, and the high rate of *Mycoplasma pneumoniae* and the low rate of influenza as CAP etiology [4, 16]. Thus, the presented data might predominantly apply to younger and healthier cohorts but should be prospectively validated in less pre-selected population-based cohorts.

However, the CAPNETZ cohort carries the advantages of a well-characterized prospective cohort with high data quality, and the relative impact of 30-day post-discharge mortality in CAPNETZ with 126 of 476 (26%) of all deaths until day 30 after discharge remains substantial.

We found several variables associated with post-discharge mortality which could be grouped into (1) demographic factors, (2) pre-existing comorbidities, (3) admission laboratory and vital parameters suggesting more severe CAP, (4) CAP-causing pathogens, and (5) other factors associated with CAP severity and treatment course. Ten risk factors both patient-related and CAP-related were identified as independently associated with early survival status after multivariable analysis. Patient-related risk factors included age, low BMI and the presence of diabetes, chronic neurological disease other than cerebrovascular diseases, and/or chronic renal diseases. Parameters correlating with severity of pneumonia included LOS, higher admission platelet counts, lower admission body temperature, the need for oxygen therapy during hospitalization, and post-obstructive pneumonia. By addition of those predictors, we calculated a score which—if validated by external cohorts—might help targeting intensified post-discharge follow-up visits in patients at risk.

To our knowledge, there are very few studies evaluating risk factors for early post-discharge mortality. One study examined 90-day post-discharge mortality in 1117 adult CAP-patients and found pre-illness functional status, comorbidities evaluated by Charlson index and CAP severity associated with that endpoint [6]. Another recent Turkish monocenter study found older age, renal comorbidity, and laboratory markers of CAP severity associated with 30-day post-discharge mortality [15]. Most other studies reporting post-discharge mortality in CAP were not designed to evaluate risk factors but to compare factors associated with in- versus post-hospital mortality [7], to evaluate prediction by clinical stability criteria [9] or to measure associations with interventions on a population based level [5, 10]. Additionally, studies on causes of readmission within 30 days after discharge identified patients with coexisting chronic comorbidities, poor functional state, and higher leucocytes or lower albumin concentrations at discharge to be readmitted more frequently [17–20].

The presence of post-obstructive CAP was the strongest single risk factor for poor outcome after discharge: Compared to 1.4% of the control group, 9.5% of the deceased cohort had post-obstructive CAP. This condition is almost always associated with a malignant neoplasm of the lung, resulting in bronchus displacement and consequent pulmonary infiltrate [21]. Post-obstructive CAP has also been shown to be associated with increased 30-day in-hospital mortality [21, 22].

Surprisingly, preexisting cardiac diseases (chronic heart failure and other chronic heart diseases) were associated with post-discharge mortality after univariable, but not after multivariable analysis in our cohort. In a recent review article, Musher et al. evaluated and summarized studies on temporal association of myocardial infarction with acute pulmonary infections [23]. Not only is the risk of myocardial infarction increased in the post-infection period, but such risk elevation persisted in the long term up to ten years after the infection. However, the absence of this association in our cohort does not exclude acute cardiovascular complications as relevant contributor to post-discharge mortality, as we are not able to provide data on acute cardiovascular complications during CAP hospitalization. Additionally our data might not apply to population-based CAP cohorts with higher age and a higher frequency of chronic comorbidities. Unfortunately, there also is no data available on the cause of post-discharge death in our study. In that context, the finding of higher thrombocyte values to be associated with post-discharge mortality is of interest. Both thrombocytosis and thrombocytopenia are associated with increased severity of pneumonia and poor outcome [24]. Activation of platelets in the course of an infectious disease can lead to increased formation of thrombi and thus may trigger cardiovascular complications such as myocardial infarction. Platelet activation markers were shown to be independent predictors for myocardial infarction as an early complication in CAP patients [25]. Another laboratory value that could be of prognostic value for early post-discharge death is troponin. Vestjens et al. showed that elevated high-sensitivity cardiac troponin T levels detected on admission in patients hospitalized with CAP has been associated with short- and long-term mortality [26]. Unfortunately, we have no data on this marker in the database.

The findings of our study carry potential implications for CAP management. Whereas in cases with data on the place of post-discharge death in our cohort 18% occurred in a nursing home, where multimorbidity and treatment restrictions might play a role, 56% of deceased patients were re-admitted to a hospital before their death, suggesting an unexpected post-hospitalization course. There is an urgent need to expand our understanding of and attitude towards the management of CAP beyond acute care, and to consider and evaluate post-discharge interventions in patients at risk. If our identified risk factors will be replicated by independent cohorts, they might enable targeted post-discharge interventions in high-risk patients like intensified follow-up visits and careful monitoring of organ function, regarding pre-existing comorbidities as well as late complications of CAP, with the aim to improve early post-discharge prognosis. A potential role of interventions to improve post-discharge prognosis after CAP has been suggested by a recent US cohort study by demonstrating an inverse association between the number of in-hospital physical and occupational therapist visits during acute care of CAP and 30-day post-discharge readmission or death rate, especially in patients with mobility limitations discharged into the community [10].

Even though the multi-national CAPNETZ study cohort is well characterized, important limitations of the study should be considered. In comparison to population-based studies, early post-discharge mortality in the prospectively recruited CAPNETZ study cohort was rather low, which might limit representiveness of our data. In addition, we missed follow-up data in 8.8% of discharged cases, which might include also deceased cases. Furthermore, laboratory values and vital signs used in the analysis were acquired at hospital admission, but no data are available on the corresponding parameters evaluated before discharge. Potential additional risk factors like data on oxygenation or acute cardiac complications during CAP could not be analyzed due to missing data. The clinical stability criteria have an established role in deciding whether a patient can be evaluated for hospital discharge [9, 11, 12], but data on these criteria were not available in our cohort. Furthermore, data on causes of death were unavailable. Finally, our proposed score to predict early post-discharge mortality needs validation within independent cohorts.

## Conclusion

In the multi-national prospective CAPNETZ study cohort, we found that early post-discharge deaths accounted for 26% of all deaths occurring until day 30 after discharge, and we identified several patient- and CAP-related risk factors associated with early post-discharge mortality. Additional studies are necessary to replicate our findings in independent cohorts and to identify and evaluate possible interventions to improve early post-discharge prognosis in CAP. Meanwhile, intensified post-discharge follow-up with close monitoring of preexisting comorbidities and CAP complications might be considered especially in elderly, multimorbid patients and those with severe CAP after hospital discharge.

## Supplementary Information

Below is the link to the electronic supplementary material.

Supplementary file1 (DOCX 48 KB)

## Data Availability

The data are stored electronically within the CAPNETZ database and are available from CAPNETZ on reasonable request.
