# Supplementary material for: Early post-discharge mortality in CAP: frequency, risk factors and a prediction tool
Source: Eur J Clin Microbiol Infect Dis. 2022 Feb 8;41(4):621–30. doi: 10.1007/s10096-022-04416-5 (PMC8934328; doi:10.1007/s10096-022-04416-5)
Supplement: Supplementary file 1 — Supplementary file1 (DOCX 48 KB) [file 10096_2022_4416_MOESM1_ESM.docx]

# Early post-discharge mortality in CAP: Frequency, risk factors and a prediction tool

Verena Glöckner, Mathias W. Pletz, Gernot Rohde, Jan Rupp, Martin Witzenrath, Grit Barten-Neiner, Martin Kolditz for the CAPNETZ Study Group

**Supplementary Data**

**e-Table 1: Performance measures of the proposed new score for prediction of 30-day post-discharge mortality for several score cut-off values**

**(bold: optimal cut-off according to Youden Index)**

+ LR: positive likelihood ratio, - LR: negative likely-hood ratio, PPV: positive predictive value, NPV: negative predictive value

| Cut-off | Patients with end point below cut-off, n (%) | Sensitivity  (95% CI) | Specificity  (95% CI) | + LR  (95% CI) | - LR  (95% CI) | PPV  (95% CI) | NPV  (95% CI) |
| --- | --- | --- | --- | --- | --- | --- | --- |
| **Proposed score (AUC (95% CI):** **0.821 (0.822 – 0.839), p-value <0.001)** | | | | | | |  |
| ≥0 | 0/144 (0.0) | 100.00  (96.7 – 100.0) | 0  (0.0 – 0.05) | 1  (1.0 – 1.0) |  | 1.5  (1.5 – 1.5) |  |
| >1 | 0/999 (0.0) | 100.00  (96.7 – 100.0) | 13.61  (12.8 – 14.4) | 1.16  (1.1 – 1.2) | 0 | 1.7  (1.7 – 1.7) | 100 |
| >2 | 3/2579 (0.1) | 97.27  (92.2 – 99.4) | 35.10  (34.0 – 36.2) | 1.50  (1.4 – 1.6) | 0.08  (0.03 – 0.2) | 2.2  (2.1 – 2.3) | 99.9  (99.6 – 100.0) |
| **>3** | **11/4480 (0.2)** | **90.00**  **(82.8 – 94.9)** | **60.89**  **(59.8 – 62.0)** | **2.30**  **(2.1 – 2.5)** | **0.16**  **(0.09 – 0.3)** | **3.3**  **(3.1 – 3.6)** | **99.8**  **(99.6 – 99.9)** |
| >4 | 38/6084 (0.6) | 65.45  (55.8 – 74.3) | 82.38  (81.5 – 83.2) | 3.72  (3.2 – 4.3) | 0.42  (0.3 – 0.5) | 5.3  (4.6 – 6.0) | 99.4  (99.2 – 99.5) |
| >5 | 69/6998 (1) | 37.27  (28.2 – 47.0) | 94.41  (93.9 – 94.9) | 6.67  (5.1 – 8.7) | 0.66  (0.6 – 0.8) | 9.1  (7.2 – 11.5) | 99  (98.9 – 99.1) |
| >6 | 93/7332 (1.3) | 15.45  (9.3 – 23.6) | 98.64  (98.3 – 98.9) | 11.34  (7.0 – 18.3) | 0.86  (0.8 – 0.9) | 14.5  (9.2 – 21.5) | 98.7  (98.6 – 98.8) |
| >7 | 107/7434 (1.4) | 2.73  (0.6 – 7.8) | 99.84  (99.7 – 99.9) | 16.68  (4.8 – 58.3) | 0.97  (0.9 – 1.0) | 20  (6.7 – 46.6) | 98.6  (98.5 – 98.6) |
| >8 | 110/7448 (1.5) | 0  (0.0 – 3.3) | 99.99  (99.9 – 100.0) | 0 | 1  (1.0 – 1.0) | 0 | 98.5  (98.5 – 98.5) |
| >9 | 110/7449 (1.5) | 0  (0.0 – 3.3) | 100  (99.9 – 100.0) |  | 1  (1.0 – 1.0) |  | 98.5  (98.5 – 98.5) |

**e-Figure 1 Frequency of the 30-day post-discharge mortality according to score values**

**e-Figure 2 Distribution of score values among the study population with n=7449 patients**
